# Less sclerotic microarchitecture pattern with increased bone resorption in glucocorticoid-associated osteonecrosis of femoral head as compared to alcohol-associated osteonecrosis of femoral head

**DOI:** 10.3389/fendo.2023.1133674

**Published:** 2023-03-08

**Authors:** Yiwei Chen, Yu Miao, Kexin Liu, Bin Zhu, Feng Xue, Junhui Yin, Jian Zou, Guangyi Li, Changqing Zhang, Yong Feng

**Affiliations:** ^1^ Department of Orthopaedics, Shanghai Sixth People's Hospital Affiliated to Shanghai Jiao Tong University School of Medicine, Shanghai, China; ^2^ Institute of Microsurgery on Extremities, Shanghai Sixth People's Hospital Affiliated to Shanghai Jiao Tong University School of Medicine, Shanghai, China; ^3^ Department of Orthopedics, The First Affiliated Hospital of Nanjing Medical University, Nanjing, China

**Keywords:** osteonecrosis of the femoral head, glucocorticoid, alcohol, micro-computed tomography, bone microstructure, bone histopathology, bone remodeling

## Abstract

**Background:**

Glucocorticoid usage and alcohol abuse are the most widely accepted risk factors for nontraumatic osteonecrosis of femoral head (ONFH). Despite distinct etiologies between glucocorticoid-associated ONFH (GONFH) and alcohol-associated ONFH (AONFH), little is known about the differences of the microarchitectural and histomorphologic characteristics between these subtypes of ONFH.

**Purposes:**

To investigate bone microarchitecture, bone remodeling activity and histomorphology characteristics of different regions in femoral heads between GONFH and AONFH.

**Methods:**

From September 2015 to October 2020, 85 patients diagnosed with GONFH and AONFH were recruited. Femoral heads were obtained after total hip replacement. Femoral head specimens were obtained from 42 patients (50 hips) with GONFH and 43 patients (50 hips) with AONFH. Micro-CT was utilized to assess the microstructure of 9 regions of interest (ROIs) in the femoral head. Along the supero-inferior orientation, the femoral head was divided into necrotic region, reactive interface, and normal region; along the medio-lateral orientation, the femoral head was divided into medial region, central region and lateral region. Decalcified and undecalcified bone histology was subsequently performed to evaluate histopathological alterations and bone remodeling levels.

**Results:**

In the necrotic region, most of the microarchitectural parameters did not differ significantly between GONFH and AONFH, whereas both the reactive interface and normal region revealed a less sclerotic microarchitecture but a higher bone remodeling level in GONFH than AONFH. Despite similar necrotic pathological manifestations, subchondral trabecular microfracture in the necrotic region was more severe and vasculature of the reactive interface was more abundant in GONFH.

**Conclusions:**

GONFH and AONFH shared similar microarchitecture and histopathological features in the necrotic region, while GONFH exhibited a less sclerotic microarchitecture and a more active bone metabolic status in both the reactive interface and normal region. These differences between GONFH and AONFH in bone microarchitectural and histopathological characteristics might contribute to the development of disease-modifying prevention strategies and treatments for ONFH, taking into etiologies.

## Introduction

Osteonecrosis of femoral head (ONFH) is known as avascular necrosis or aseptic necrosis of the femoral head, associated with an interruption of the blood supply (1). One of the pathological features of ONFH is the damage of subchondral vascular microcirculation, which leads to osteonecrosis, eventually resulting in repeated microfractures in the femoral head and articular collapse (2).There is a substantial risk that intractable pain, femoral head collapse, and end-stage osteoarthritis will occur, ultimately necessitating total hip arthroplasty (THA). Annually, between 10,000 and 20,000 new cases of the disease are diagnosed in the United States (3). The cumulative number of Chinese patients with nontraumatic ONFH was estimated to be roughly 8.12 million in a recent nationally representative survey (4).

The etiology and pathogenesis of nontraumatic ONFH are not well understood (5). Glucocorticoid usage and alcohol abuse are the most widely reported risk factors for nontraumatic ONFH (6). More than 80% of patients with nontraumatic ONFH are thought to be caused by glucocorticoid usage and excessive alcohol intake (7–9). Glucocorticoid use and alcohol abuse may lead to the deterioration of bone tissue in the femoral head *via* direct or indirect harmful effects, with the potential mechanism being vascular damage, increase in intraosseous pressure, intraosseous coagulation, disruption of osteogenic differentiation, and mechanical stresses (10–12). In addition, glucocorticoid has the potential effect on directing precursor cells towards an adipogenic or osteogenic pathway (13). There is a hypothesis that abnormal alcohol metabolism may lead to the deterioration of bone tissue in the femoral head through toxic by-products such as acetaldehyde, free radicals and acetaldehyde adducts (14), with the potential mechanism of intravascular coagulation and coagulation cascade regulation (15). Alcohol could also disrupt osteogenic differentiation and promotes lipogenesis of bone marrow mesenchymal stem cells, in the pathogenesis of alcohol-associated osteonecrosis of femoral head (AONFH) (10, 11). A few investigations have revealed the presence of glucocorticoid-associated ONFH (GONFH) and/or alcohol-associated ONFH (AONFH) in bone histopathological alterations. Chernetsky et al. discovered that after bone death, histologic abnormalities of necrosis and repair evolved in a predetermined order, with the GONFH exhibiting a more rapid progression (16). According to Kim et al.’s histological investigation, patients with AONFH or idiopathic ONFH had normal or almost normal cancellous bone in the acetabulum and proximal femur, whereas GONFH may be linked to more extensive osteonecrosis (17). In a study by Wei et al., GONFH was characterized by multiple “osteolytic bone destruction”, while AONFH was manifested by some kinds of “coagulative destruction” (18, 19). When compared to age-matched controls, patients with ONFH brought on by glucocorticoid usage or sickle-cell illness have been observed to have worse THA outcomes in clinical settings (20–22). This heterogeneity could be explained by the various biological impacts that various risk factors have on the bone tissue.

Despite distinct etiologies between GONFH and AONFH, the differences in bone microarchitecture and histopathology between these two major nontraumatic ONFH subtypes are infrequently explored. Bone microarchitectural and histopathological analysis may increase our understanding of the pathogenesis and progression of ONFH, which may lead to the development of disease-modifying prevention strategies and therapies for ONFH, taking into account its etiologies. We therefore propose that the bone microarchitectural and histopathological features of the femoral head should differ between GONFH and AONFH. In order to address this, we evaluated microarchitecture, bone remodeling activity and histopathological alterations of trabecular bone in different regions of the femoral head based on the three-pillar system (23–25) from patients with GONFH and AONFH, using micro-CT, decalcified and undecalcified bone histology.

## Materials and methods

### Subjects

Between September 2015 and October 2020, 85 patients diagnosed with ONFH who underwent THA surgery, were recruited in the study. These patients who participated in this study were divided into two groups based upon which etiology they belonged to (1): GONFH or (2) AONFH. Patients were recruited to the GONFH group if they met the classification criteria of GONFH included the following: (1) Patients should have a history of glucocorticoid use more than 2 g of prednisolone or its equivalent within 3 months;(2) ONFH was diagnosed within 2 years after glucocorticoid administration;(3) There were no other risk factors except glucocorticoids (26). A total of 85 patients (100 hips) diagnosed with ONFH were recruited in the study. 42 GONFH patients (50 hips) had various indications for glucocorticoid treatment, including autoimmune diseases [systemic lupus erythematosus (n=15), rheumatoid arthritis (n=2)], renal diseases [nephritic syndrome (n=5), pyelonephritis (n=1), IgA nephropathy (n=2), chronic nephritis (n=2) and renal transplantation (n=1)], dermatogic diseases [psoriasis (n=1)), skin pruritus (n=1), eczema (n=2), urticaria (n=1), and neurodermatitis (n=2)], thrombocytopenic purpura (n=2), chronic pulmonary fibrosis (n=1), craniopharyngioma (n=1), myocarditis (n=1), bronchial asthma (n=2).

Regarding AONFH, we collected information about age at starting and cessation of alcohol consumption, usual frequency of drinking, and the usual volume of alcohol intake by beverage type. This volume was consequently converted to grams of ethanol and values for each beverage type were added. The ethanol content for calculation was as follows: 4.5% for beer, 12% for wine, 43% for whisky, 15.5% for rice wine and 50% for baijiu (a distilled alcoholic beverage made in China) (27). We described habitual drinking in patients with AONFH as more than 3032 drink years of the cumulative alcohol intake according to the previous report (27). The cumulative drinking amount termed as ‘drink-years’ was calculated by multiplying the weekly ethanol consumption (gram) by the total number of years of drinking. Patients recruited to the AONFH group should also meet classification criteria of AONFH: (1) patients should have a history of alcohol intake of more than 400ml/week of pure ethanol (320g/week of any type of alcoholic beverage) for more than 6 months;(2) ONFH was diagnosed within 1 year after this dose of alcohol intake;(3) There should be no other risk factors (28). 43 participants (50 hips) with AONFH were classified into the AONFH group. Patients who were diagnosed with bilateral ONFH are all at different stages.

GONFH and AONFH subjects were well matched on demographic factors and medical conditions (**Table 1**), with the exception being the proportion of males, which was significantly higher in the AONFH group than that in the GONFH group (P<0.0001).

**Table 1:** Characteristics of the study participants.

	GONFH	AONFH	P-value
Characteristics of study participants			
Number of patients	42	43	
Gender (male/female)	22/20	42/1	**<0.0001**
Age (years)	47.29(34.75-45)	51.44(43-58)	0.1610
BMI (kg/m^2^)	24.17(22.42-26.51)	24.83(22.65-27.56)	0.5187
Smoker n (%)	19(45.23)	26(60.47)	0.1597
Diabetes	4	4	0.9721
Hypertension	8	9	0.8283
Characteristics of osteonecrosis			
Number of hips	50	50	
Side (right: left)	24:26	20:30	0.5459
Timing since the onset of the ONFH, months	27.05(8-18)	24.12(8-12)	0.8168
Glucocorticoid dosage			
Glucocorticoid pulse therapy, n (%)	14(33)	NA	NA
Duration of glucocorticoid administration, years	9.12(7-10)	NA	NA
Glucocorticoid treatment			
Drug species	Prednisone	NA	NA
	Methylprednisolone	NA	NA
	Dexamethasone	NA	NA
Alcohol consumption			
Duration of alcohol consumption	NA	23(20-30)	NA
Weekly ethanol consumption (g/week)	NA	587.3(525-542.5)	NA
Ethanol drink-years ((g/week) × years)	NA	13142(7875-16275)	NA
ARCO STAGE			0.9382
STAGE II	8	9	
STAGE IIIA	12	14	
STAGE IIIB	17	16	
STAGE IV	13	11	
Japanese classification system			0.8863
Type A	1	2	
Type B	6	6	
Type C1	22	19	
Type C2	21	23	

Data are the median (interquartile range) for quantitative variables and no. (%) of patients for qualitative variables. Bold indicates statistically significant difference. NA, Not Available.

**Figure 1:**
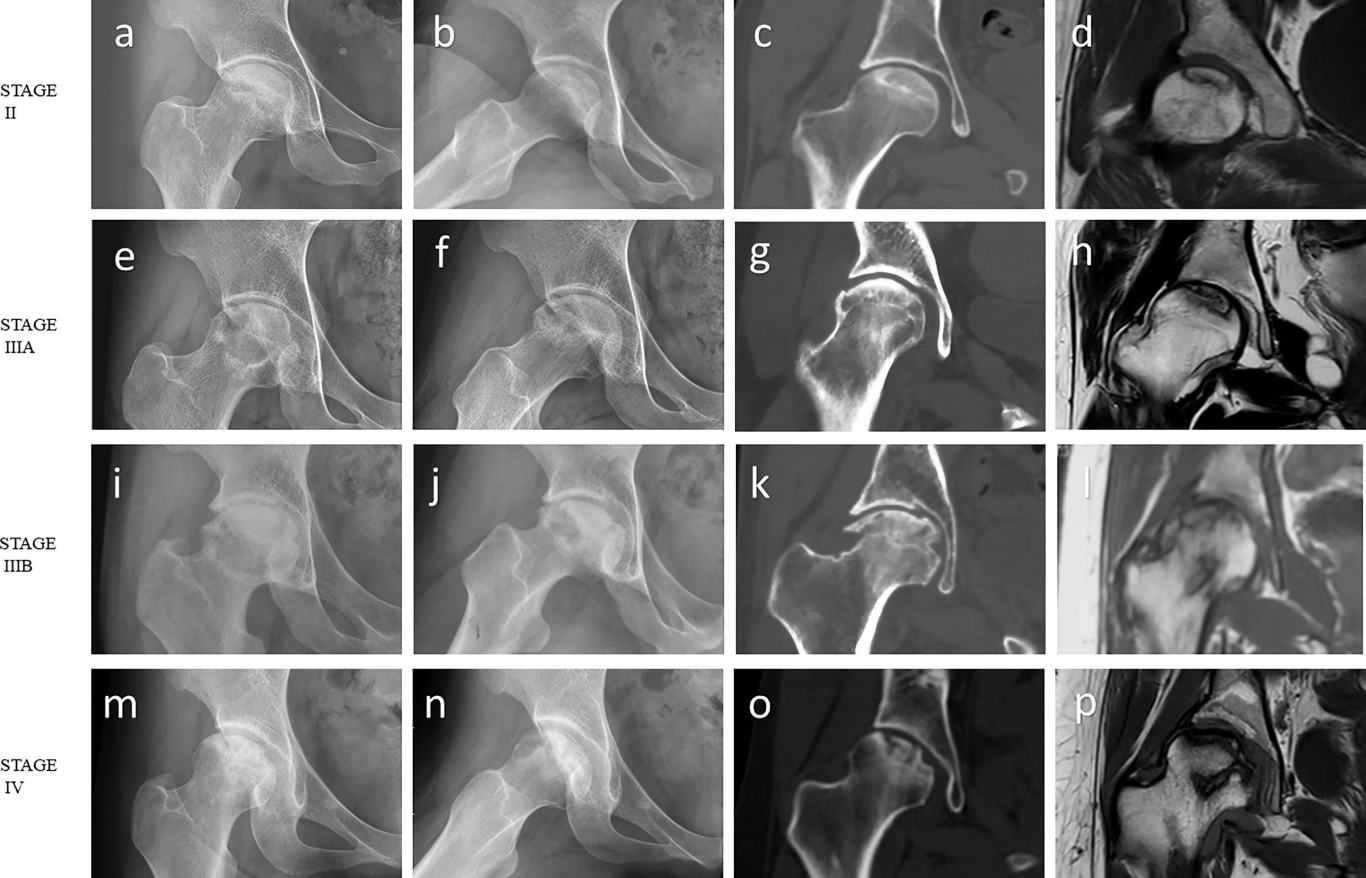
Imaging features of ONFH. **(A-D)** ARCO stage II: The radiograph showed ﻿focal osteoporosis and osteosclerosis in the femoral head. However, there were no signs of collapse, and the femoral head remained spherical on the coronal CT image and T1-weighted MR image. **(E-H)** ARCO stage IIIA: Subchondral fracture and ﻿flattening of the femoral head was observed on the radiograph or coronal CT image. Femoral head depression was less than 2 mm. **(I-L)** ARCO stage IIIB: Femoral head depression was more than 2 mm. **(M-P)** ARCO stage IV: Osteoarthritis of the hip joint with joint space narrowing was detected on the radiograph. Coronal CT image and T1-weighted MR image showed advanced degenerative alterations and entire joint destruction. Note: **(A, E, I, M)** ﻿Frontal radiograph; **(B, F, J, N)** Frog-leg lateral radiograph; **(C, G, K, O)** Coronal CT image; **(D, H, L, P)** Coronal T1-weighted MR image.

Exclusion criteria for ONFH patients were as follows: (1) Other recognized metabolic or bone conditions, such as thyroid or parathyroid illness, and malignancy, that may impact bone metabolism; (2) undergoing additional medications that influence bone metabolism such as anti-resorptive drugs, calcitonin, thyroid or parathyroid hormone therapy, or hormonal replacement therapy; or (3) history of hip fracture and osteotomy (29–31). Information about glucocorticoid usage, alcohol consumption, smoking in two groups is shown in **Table 1**. All cases were classified with different clinical stages recommended by the Association Research Circulation Osseous (ARCO) (32). The cases in this study were all ARCO stage II, IIIA, IIIB or IV, with severe pain or severe hip dysfunction (**Figure 1**). Given that location of the necrosis may affect topographical observations, the Japanese classification is important to understand the specific changes, particularly addressing lateral extension (33). Therefore, we use the Japanese Investigation Committee (JIC) system to classify all cases as well. Type A lesions account for only a third or less of the weight-bearing portion. Type B lesions accounted for two-thirds or less of the weight-bearing portion. Both type C1 and C2 lesions account for more than two thirds of the medial weight bearing portion of the acetabulum, but C2 lesions extend laterally to the acetabulum margin, while C1 lesions do not (33). Informed consent was obtained from each patient. The study protocol was approved by the Human Research Ethics committee of our hospital and complied with the Declaration of Helsinki.

### Micro-CT examination

Following THA surgery, specimens of the femoral head were collected and preserved in a -80°C freezer. Then, they were scanned using the high resolution micro-CT system (μCT100, Scanco Medical, Bassersdorf, Switzerland), with the following scan settings: X-ray tube voltage, 70 kVp; tube current, 200 μAs; exposure time, 300 ms; projection number, 1000; and voxel size, 73.6 μm. The scan length was approximately 49 mm, resulting in 1200 slices and a 45-minute scan time.

**Figure 2:**
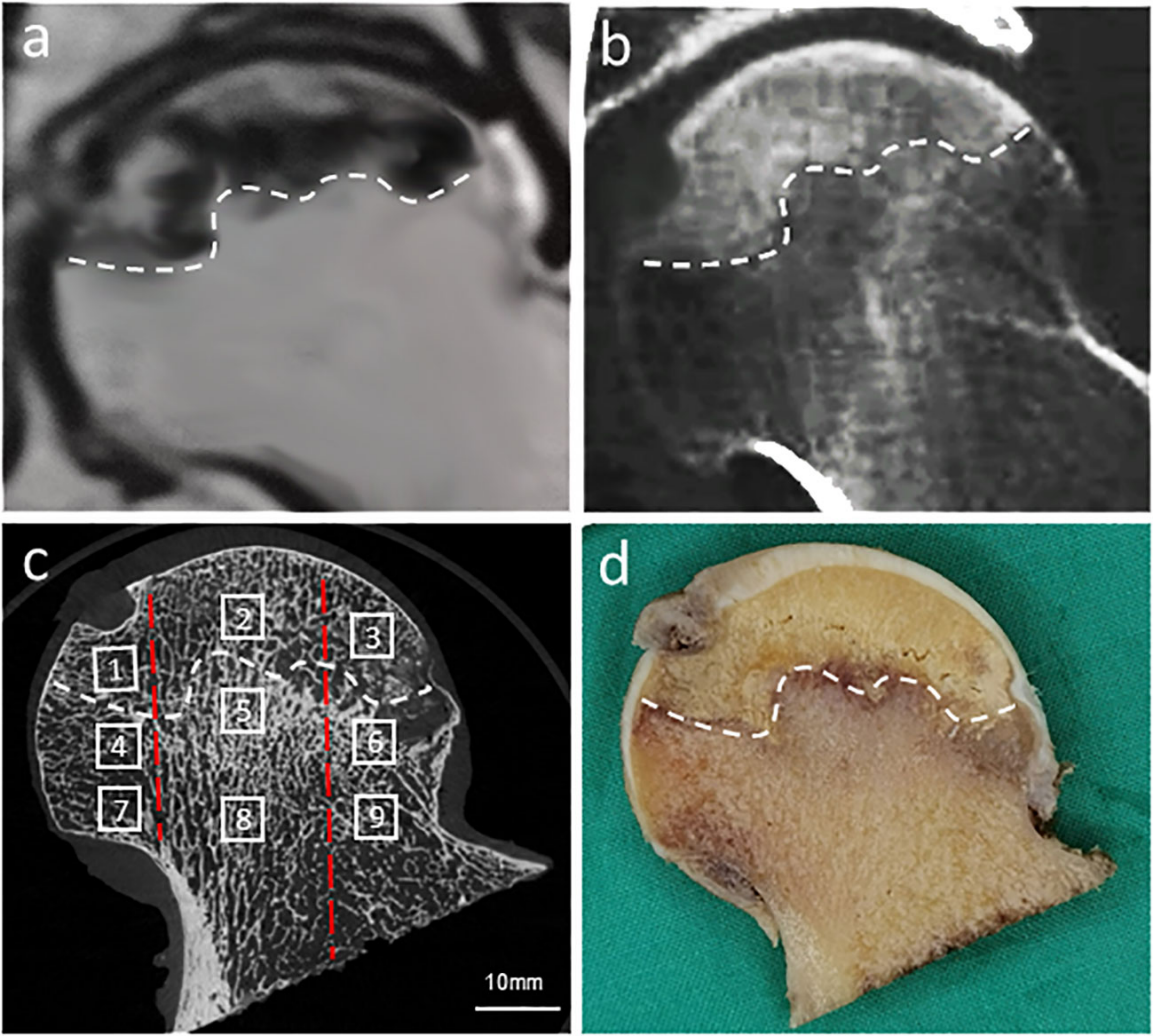
Location, orientation and segmentation of different regions of interest (ROIs) in the femoral head. A high degree of consistency was demonstrated between MR image **(A)**, CT image **(B)**, corresponding micro-CT image **(C)**, and gross examination of the coronal plane of the femoral head **(D)**. In each femoral head, 9 ROIs were set in the necrotic region (1, Sup-Med; 2, Sup-Cen; 3, Sup-Lat), reactive interface (4, Cen-Med; 5, Cen-Cen; 6, Cen-Lat), and normal region (7, Inf-Med; 8, Inf-Cen; 9, Inf-Lat). The white dotted line indicated the reactive interface, while the two red dotted lines divided the femoral head into three regions in the medio-lateral orientation: medial region, central region, and lateral region based on the CJFH three-pillar system.

Orientation adjustment of the femoral head, segmentation of the regions of interest (ROIs), and measurement of bone microstructural parameters in each region were performed by the built-in software. Orientations of all femoral heads were adjusted based on anatomical landmarks using the fovea capitis femoris and the three−pillar structure theory (34, 35). The reactive interface (or necrotic-normal interface) was comprehensively determined by the Magnetic Resonance (MR) Imaging, CT and micro-CT imaging, and gross examination of the coronal plane of the femoral head specimen. 9 ROIs were set in the coronal plane of the femoral head (**Figure 2**). Specifically, along the supero-inferior orientation, the femoral head was divided into three main regions with reference to the boundary of the necrotic lesion: superior region (necrotic region), central region (i.e. reactive interface or necrotic-normal junction region), and inferior region (normal region); along the medio-lateral orientation, the femoral head was divided into another three main regions with reference to the China-Japan Friendship Hospital (CJFH) three−pillar system: medial region, central region and lateral region (23–25). The ROIs of reactive interface were routinely located 0.5 mm from the boundary of the necrotic lesion. The inferior region (normal region) is located close to the base of the femoral head, which is at a distance from the central region (reactive interface). Thus, a total of nine ROIs (Sup–Med, Sup–Cen, Sup–Lat, Cen–Med, Cen–Cen, Cen–Lat, Inf–Med, Inf–Cen, and Inf–Lat) were included in the microstructure analysis. For the specimens in which the lateral boundary of the necrotic lesion was medial to the central pillar, relative lateral ROIs were not included for analysis. In each ROI, a cylindrical bone specimen (5 mm in height and 5 mm in diameter) was set for analysis.

**Figure 3:**
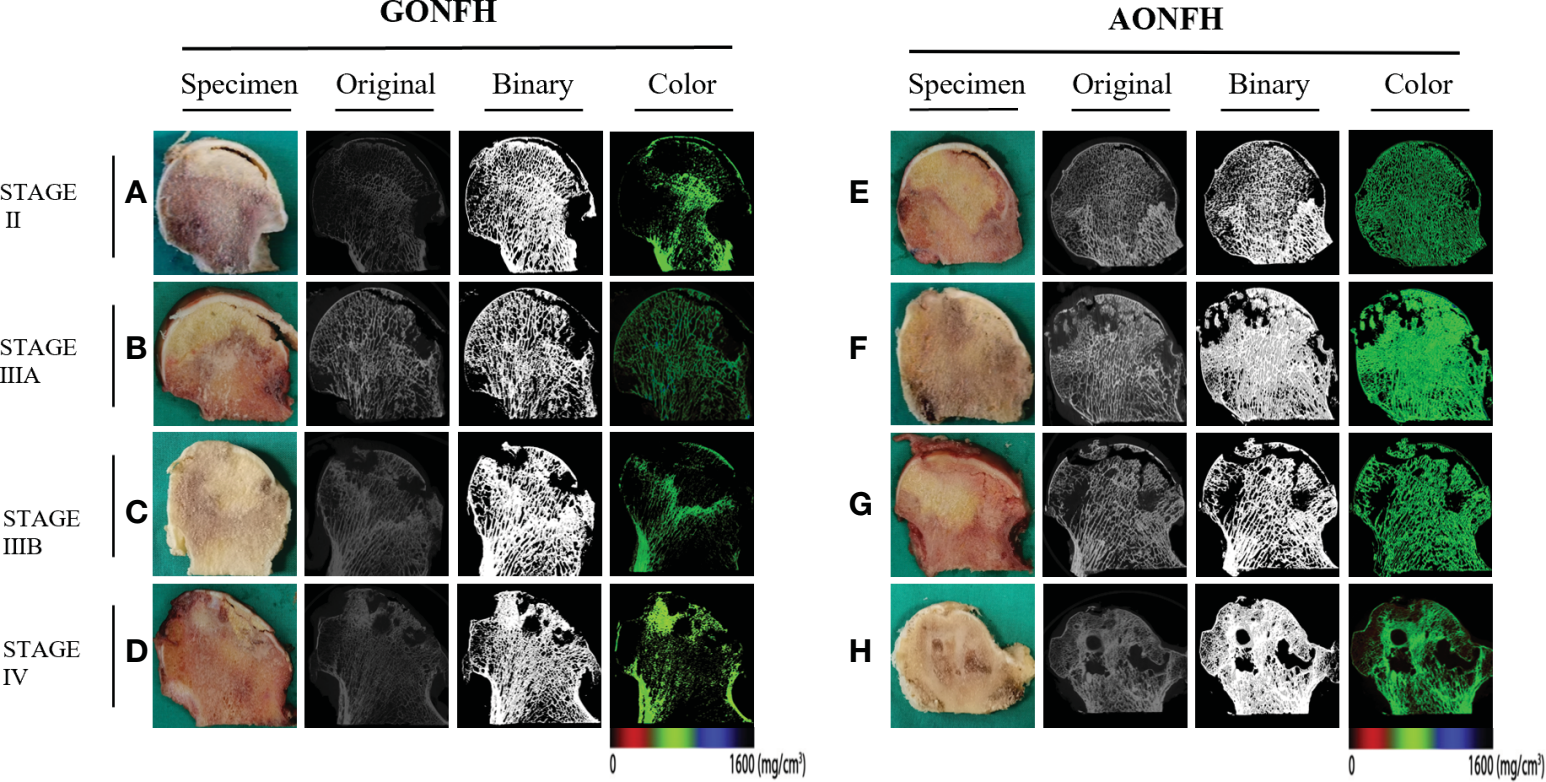
﻿Representative original, binary and color micro-CT images corresponding to the coronally sectioned gross specimen of the femoral head from patients with GONFH **(A-D)** and patients with AONFH **(E-H)**. **(A, E)** ARCO stage II. **(B, F)** ARCO stage IIIA. **(C, G)** ARCO stage IIIB. **(D, H)** ARCO stage IV. Note: The color images represented mineralization distribution in trabecular bone. Red, green and blue represent low, intermediate and high mineral density, respectively.

**Figure 4:**
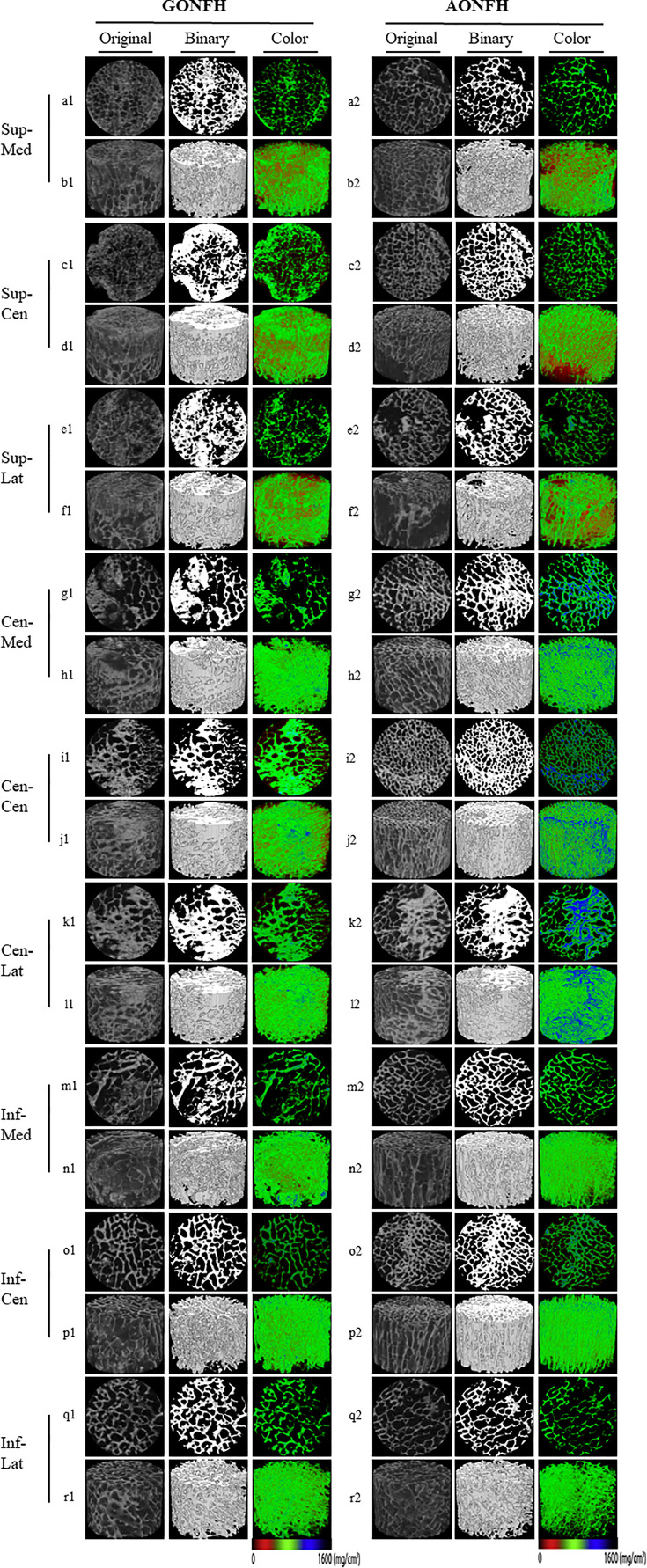
Representative original, binary and color micro-CT images of different ROIs in the femoral head from patients with GONFH (a1-r1) and patients with AONFH (a2-r2). 2D visualization of the cross-section of the Sup-Med (a1, a2) , Sup-Cen (c1, c2) , Sup-Lat (e1, e2) , Cen-Med (g1, g2) , Cen-Cen (i1, i2) , Cen-Lat (k1, k2) , Inf-Med (m1, m2), Inf-Cen (o1, o2), and Inf-Lat (q1, q2); 3D reconstruction of the Sup-Med (b1, b2) , Sup-Cen (d1, d2) , Sup-Lat (f1, f2), Cen-Med (h1, h2) , Cen-Cen (j1, j2) , Cen-Lat (l1, l2) , Inf-Med (n1, n2), Inf-Cen (p1, p2), and Inf-Lat (r1, r2). The color images represented mineralization distribution in trabecular bone. Red, green and blue represent low, intermediate and high mineral density, respectively.

Following scanning and reconstruction, the pictures were converted to binary images using a fixed threshold. To distinguish between soft tissue and calcified tissue, a consistent global threshold range (90 to 255) based on grayscale histogram analysis and empirical observations was applied (36, 37). Color images were also produced based on X-ray attenuation coefficient values, representing mineralization distribution in the trabeculae (**Figures 3**, **4**). Bone microarchitecture parameters were calculated. In specimens with cystic lesions and patchy necrotic debris, the measurement of bone microarchitecture was limited to the trabecular region, where cysts and debris were not present.

The following microarchitectural parameters were measured: bone volume fraction (BV/TV) (%), bone surface/volume ratio (BS/BV) (1/mm), trabecular thickness (Tb.Th) (μm), trabecular separation (Tb.Sp) (μm), trabecular number (Tb.N) (1/mm), structure model index (SMI), degree of anisotropy (DA), connectivity density (Conn.D) (1/mm3) (38).

### Specimen preparation

After micro-CT scanning, all femoral heads were cut in half with a diamond saw, coronally separating the anterior hemisphere from the posterior hemisphere (**Figures 3A-C**). The characteristics of MR Imaging, CT and micro-CT imaging corresponding to each gross specimen were evaluated rigorously to determine the location, extent, and boundary of the necrotic lesion. A 10-mm-thick coronal bone slice was then sectioned from each femoral head hemisphere. One slice was prepared for decalcified bone sectioning, and the other was utilized for undecalcified bone histology. Each slice was divided into nine ROIs as aforementioned. All of these specimens were preserved in paraformaldehyde for two weeks, followed by the corresponding procedure for histology without or with calcification. Samples were embedded in paraffin wax, sectioned into 5-m-thick slices, and stained with H&E after being decalcified. Undecalcified samples were embedded in methyl-methacrylate, sectioned into 5-μm-thick slices, and were stained using the Goldner’s Trichrome technique.

### Histopathological assessment and histomorphometry

The decalcified sections were examined for the evidence of osteonecrosis and secondary pathologic changes. The microfracture incidence was calculated as the ratio of the number of hips with microfracture/total number of hips. The patchy debris incidence was calculated as the ratio of the number of hips with patchy debris/total number of hips. Quantifications on angiogenesis were performed on the decalcified sections. Vessel number was counted in ten randomly selected visual fields (×200 magnification) in the medial, central, and lateral reactive interfaces, respectively. The evaluation was performed by two independent researchers who were blind to the identity of group.

For the undecalcified sections, histomorphometry was performed using Bioquant Osteo Histomorphometry software (Bioquant Osteo, Nashville, TN, USA). The following bone formation and resorption parameters were measured in each ROI: thickness of osteoid (O.Th, μm), percentage osteoid volume (OV/BV) (%), percentage osteoid surface (OS/BS) (%), specific osteoid surface (OS/BV) (mm^2^/mm^3^), percentage eroded surface (ES/BS) (%), specific eroded surface (ES/BV) (mm^2^/mm^3^) and eroded surface in bone tissue volume (ES/TV) (mm^2^/mm^3^) (39).

### Statistical analysis

We randomly selected 10 samples and assessed the inter-observer and intra-observer agreements using Root Mean Square Error Estimation (RMSE). Two experienced observers determined the ROIs of selected samples independently. We reported the RMSE of the difference between two observers for each region stratified by subtype of ONFH. The results showed good intra and inter-observer reproducibility in different regions (**Supplementary Table 1**).

The Kolmogorov-Smirnov test was used to determine if parameters followed a normal (Gaussian-shaped) distribution and no significant departures were identified. The unpaired Student’s t test was used to compare the microarchitecture parameters, vessel number, and bone remodeling parameters between groups. A conservative two-tailed P < 0.05 was chosen *a priori* to declare a statistically significant result to account for multiple comparisons and to avoid type I errors. Analysis of data was performed using the Statistics Package for Social Sciences (SPSS for Windows, version 17.0; SPSS Inc, Chicago, IL, USA). Continuous data are presented as mean ± standard deviation (SD). Gender, smoker, diabetes, hypertension, side of ONFH, ARCO stage (40), JIC stage, the microfracture incidence, and the patchy debris incidence were compared between GONFH and AONFH groups using Fisher’s exact test, Chi square or Chi square test with Yates correction, as appropriate.

## Results

### Comparative analysis of bone microarchitecture between GONFH and AONFH

In Sup-Med region, none of the microarchitecture parameters differed significantly between GONFH and AONFH, with the exception of DA. There were significantly lower values of DA in GONFH, compared to AONFH. Similarly, in Sup-Cen region, none of the microarchitecture parameters differed significantly between GONFH and AONFH, with the exception of SMI (**Figure 4**; **Table 2**). There were significantly higher values of SMI in GONFH, compared to AONFH. In Sup-Lat region, there were lower values of Tb.N, higher values of DA in GONFH than in AONFH, while the other parameters showed no significant difference.

**Table 2:** Comparison of microarchitecture parameters in different regions between GONFH and AONFH.

Region	Variables	GONFH	AONFH	P	Region	Variables	GONFH	AONFH	P	Region	Variables	GONFH	AONFH	P
Sup-Med	BV/TV (%)	25.59±9.55	27.57±13.32	0.3960	Cen-Med	BV/TV (%)	41.99±18.09	49.01±15.96	**0.0425**	Inf-Med	BV/TV (%)	17.48±6.25	19.50±9.90	0.2728
	BS/BV(1/mm)	12.88±3.35	13.03±3.57	0.8282		BS/BV(1/mm)	9.39±3.64	8.38±2.95	0.1314		BS/BV(1/mm)	15.11±3.30	14.48±3.85	0.3807
	Tb.Th (μm)	312.10±88.25	297.80±86.17	0.3482		Tb.Th (μm)	461.60±269.51	450.02±173.80	0.5994		Tb.Th (μm)	240.70±55.33	264.04±66.29	0.0992
	Tb.N(1/mm)	0.83±0.24	0.90±0.28	0.1676		Tb.N(1/mm)	0.95±0.25	1.12±0.23	**0.0008**		Tb.N(1/mm)	0.64±0.21	0.71±0.23	0.1105
	Tb.Sp (μm)	792.30±253.01	778.30±284.40	0.4316		Tb.Sp (μm)	694.80±329.31	566.10±114.91	**0.0087**		Tb.Sp (μm)	918.40±165.01	906.02±194.10	0.4944
	Conn.D (1/mm^3^)	3.45±1.39	3.48±1.37	0.9090		Conn.D (1/mm^3^)	2.92±1.49	2.99±1.26	0.5982		Conn.D (1/mm^3^)	2.32±0.83	2.32±0.95	0.9947
	SMI	1.59±0.55	1.35±0.76	0.2118		SMI	0.675±1.10	0.18±1.01	**0.0043**		SMI	1.78±0.36	1.61±0.46	**0.0390**
	DA	1.81±0.29	1.94±0.33	**0.0373**		DA	1.77±0.35	1.78±0.36	0.9716		DA	2.28±0.50	2.39±0.61	0.3153
Sup-Cen	BV/TV (%)	30.02±10.86	33.77±12.26	0.1108	Cen-Cen	BV/TV (%)	49.01±16.08	62.18±13.23	**<0.0001**	Inf-Cen	BV/TV (%)	34.29±8.80	37.10±11.55	0.1613
	BS/BV(1/mm)	11.97±2.96	11.25±2.76	0.2104		BS/BV(1/mm)	8.75±3.27	6.73±2.02	**0.0004**		BS/BV(1/mm)	12.10±3.49	10.89±3.08	0.0694
	Tb.Th (μm)	323.01±103.30	329.80±85.92	0.5075		Tb.Th (μm)	475.70±295.71	519.81±183.50	**0.0014**		Tb.Th (μm)	301.90±85.77	327.10±94.96	0.1272
	Tb.N(1/mm)	0.99±0.24	1.03±0.26	0.0902		Tb.N(1/mm)	1.13±0.22	1.25±0.20	**0.0055**		Tb.N(1/mm)	1.07±0.14	1.14±0.19	0.0939
	Tb.Sp (μm)	726.70±288.81	691.40±231.71	0.7083		Tb.Sp (μm)	549.90±105.21	456.01±92.71	**<0.0001**		Tb.Sp (μm)	642.00±81.16	616.60±99.07	0.1650
	Conn.D (1/mm^3^)	3.50±1.42	3.50±1.38	0.9863		Conn.D (1/mm^3^)	3.34±1.83	2.65±1.35	**0.0354**		Conn.D (1/mm^3^)	3.13±1.31	3.34±1.53	0.5287
	SMI	1.29±0.62	1.04±0.89	**0.0244**		SMI	0.27±1.11	-0.70±1.35	**<0.0001**		SMI	0.99±0.60	0.73±0.59	**0.0360**
	DA	1.88±0.40	1.80±0.33	0.5298		DA	1.80±0.33	1.77±0.38	0.3343		DA	2.51±0.47	2.52±0.44	0.5852
Sup-Lat	BV/TV (%)	30.22±11.43	34.47±10.05	0.0726	Cen-Lat	BV/TV (%)	47.15±18.56	54.07±14.26	**0.0394**	Inf-Lat	BV/TV (%)	17.27±7.90	20.26±8.50	0.0564
	BS/BV(1/mm)	12.14±3.23	11.02±2.73	0.0841		BS/BV(1/mm)	8.70±3.65	7.46±2.39	**0.0478**		BS/BV(1/mm)	15.99±4.25	15.01±3.65	0.2151
	Tb.Th (μm)	330.64±113.25	344.281±69.17	0.5040		Tb.Th (μm)	472.20±219.61	490.31±174.30	0.2639		Tb.Th (μm)	243.70±69.29	249.03±54.51	0.6669
	Tb.N(1/mm)	0.88±0.28	1.01±0.20	**0.0145**		Tb.N(1/mm)	1.03±0.23	1.14±0.18	**0.0071**		Tb.N(1/mm)	0.74±0.15	0.79±0.22	0.2244
	Tb.Sp (μm)	739.30±225.70	704.30±193.50	0.5089		Tb.Sp (μm)	625.20±296.21	546.21±110.62	**0.0366**		Tb.Sp (μm)	886.70±153.50	828.10±155.91	0.1285
	Conn.D (1/mm^3^)	3.35±1.48	3.18±1.11	0.7346		Conn.D (1/mm^3^)	2.84±1.35	2.64±1.19	0.4336		Conn.D (1/mm^3^)	2.58±0.84	2.90±1.27	0.4815
	SMI	1.49±0.57	1.30±0.78	0.1976		SMI	0.25±1.33	-0.12±1.19	0.0640		SMI	1.67±0.39	1.55±0.40	0.1785
	DA	1.83±0.38	1.71±0.28	**0.0471**		DA	1.78±0.37	1.78±0.34	0.9658		DA	2.26±0.60	2.15±0.60	0.3736

Results are expressed as mean ± SD. Bold indicates statistically significant difference.

In Cen-Med region (**Figure 4**; **Table 2**), BS/BV, Tb.Th, Conn.D and DA were not significantly different between GONFH and AONFH. However, there were significantly lower values of BV/TV and Tb.N, higher values of Tb.Sp and SMI in GONFH, compared to AONFH.

In Cen-Cen region, all the microarchitecture parameters were significantly different between GONFH and AONFH, with the exception of DA (**Figure 4**; **Table 2**). Compared to AONFH, there were significantly higher values of BS/BV, Tb.Sp, Conn.D and SMI in GONFH, with significantly lower values of BV/TV, Tb.Th, Tb.N.

In Cen-Lat region, Tb.Th, Conn.D, SMI and DA were not significantly different between GONFH and AONFH (**Figure 4**; **Table 2**). Nevertheless, there were significantly lower values of BV/TV and Tb.N, higher values of BS/BV and Tb.Sp in GONFH than in AONFH.

In Inf-Med region, there were higher values of SMI in GONFH than in AONFH, while the other parameters showed no significant difference. In Inf-Cen region, BV/TV, BS/BV, Tb.Th, Tb.N, Tb.Sp, Conn.D and DA were not significantly different between GONFH and AONFH. However, there were significantly higher values of SMI in GONFH, compared to AONFH. In Inf-Lat region, none of the microarchitecture parameters differed significantly between GONFH and AONFH (**Figure 4**, **Table 2**).

### Comparative analysis of bone histopathology between GONFH and AONFH

**Figure 5:**
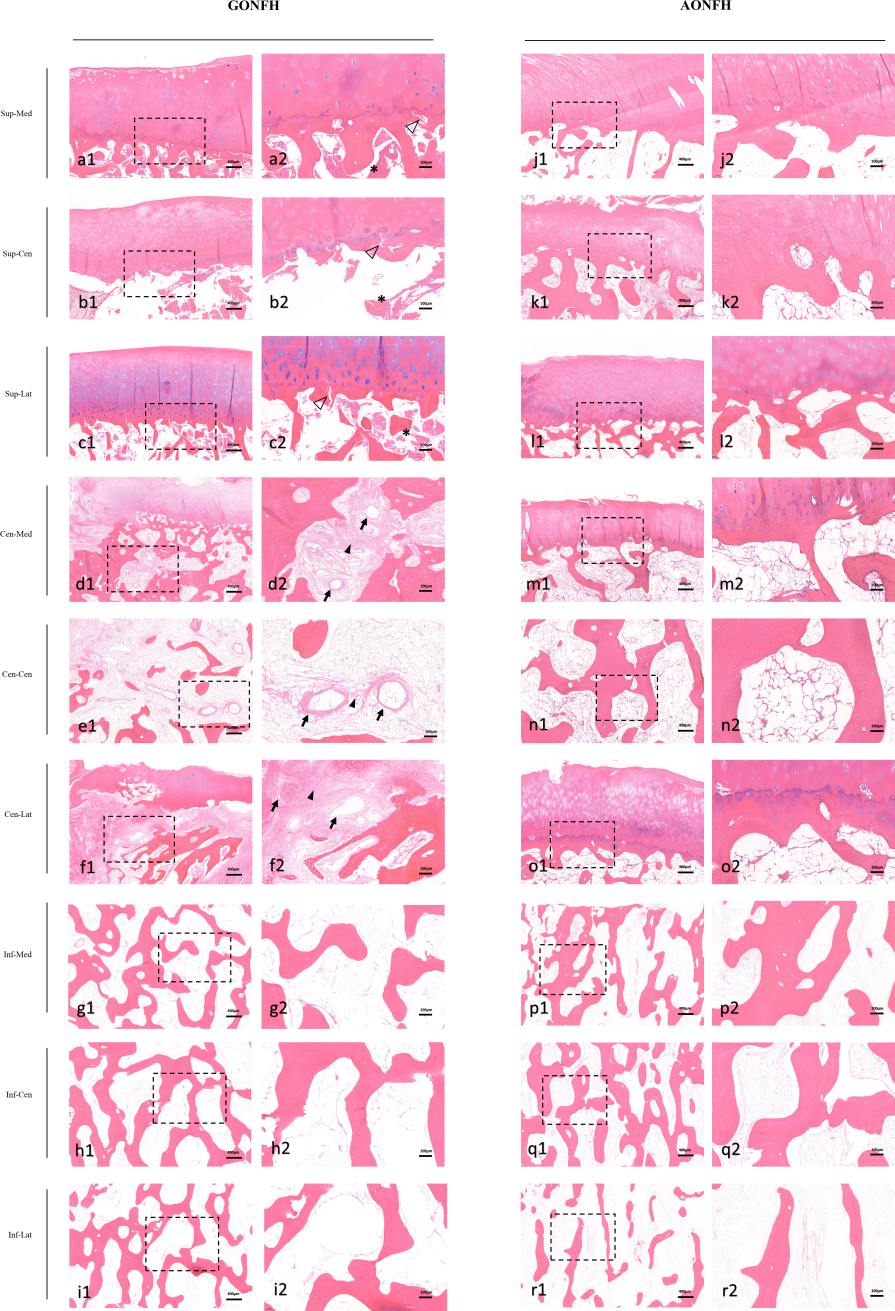
Photomicrographs of decalcified sections of different ROIs in the femoral head from patients with GONFH **(A-I)** and patients with AONFH **(J-R)**. The decalcified sections exhibit similar characteristics to those seen in imaging and typical ONFH appearances, with the patchy debris (*), microfracture (hollow arrows), vascular-rich granulation tissue (solid arrows) and reparative fibrovascular tissue (arrowheads). Stain: hematoxylin and eosin (HE); (a1-r1) magnification: ×40; (a2-r2) magnification: ×100.

In the necrotic region of the femoral head, bone marrow necrosis and osteocytic death were observed in both groups. Hematopoietic cells disappeared and adipose nucleus staining was absent in the bone marrow, indicating the necrosis of bone marrow (**Figure 5**). The necrotic lesions were not scattered and patchy in the bone marrow space, but exhibiting as a large continuous area with different shapes. In addition, most bone trabeculae represented empty osteocyte lacunae, seldom encompassing some pycnotic nuclei of osteocytes. In the subchondral trabecular bone of the necrotic region, microfracture and patchy debris was frequently observed, which was more obvious and severe in GONFH. In the medial necrotic region, the microfracture incidence was 68% (34/50) in the GONFH group and 44% (22/50) in the AONFH group; the patchy debris incidence was 74% (37/50) in the GONFH group and 52% (26/50) in the AONFH group. In the central necrotic region, the microfracture incidence was 72% (36/50) in the GONFH group and 52% (26/50) in the AONFH group; the patchy debris incidence was 82% (41/50) in the GONFH group and 62% (31/50) in the AONFH group. In the lateral necrotic region, the microfracture incidence was 86% (43/50) in the GONFH group and 62% (31/50) in the AONFH group; the patchy debris incidence was 90% (45/50) in the GONFH group and 72% (36/50) in the AONFH group. (**Supplementary Figure 1**). Chi-square test showed that the microfracture incidence and patchy debris incidence in the GONFH group was significantly higher than that of the AONFH group (P < 0.05).

In the necrotic-normal junction region, reparative process, such as vascular-rich granulation tissue, was observed. Histiocytes and giant cells aggregated in the reactive fibrous interface, especially in the lateral aspect. Although vessels penetrated into the fibrotic capsule, the angiogenesis was blocked at the boundary of the sequestrum and reparative fibrovascular tissue could not penetrate into the bone marrow space of the necrotic region. Constantly, the reparative fibrovascular tissue would convert to a sclerotic rim in the late stages of the disease, which was attributed to the bone formation by osteoblasts.

In the medial reactive interface, the vessel number was 8.76 ± 3.47 in the GONFH group and 4.90 ± 2.03 in the AONFH group. In the central reactive interface, the vessel number was 9.74 ± 3.33 in the GONFH group and 5.72 ± 1.72 in the AONFH group. In the lateral reactive interface, the vessel number was 10.72 ± 3.94 in the GONFH group and 6.82 ± 2.27 in the AONFH group. There were significant statistical differences in the vessel number between the two groups. Accordingly, the angiogenesis was more abundant in the reactive interface in GONFH, bringing about a more active bone remodeling status, despite the failure of thorough reparative substitution of the sequestrum in both groups (**Figure 5**; **Supplementary Figure 1**).

### Comparative analysis of bone remodeling levels between GONFH and AONFH

Due to the devitalization status of the necrotic region, the Sup–Med, Sup–Cen and Sup–Lat regions were not analyzed in the aspect of bone remodeling levels.

**Figure 6:**
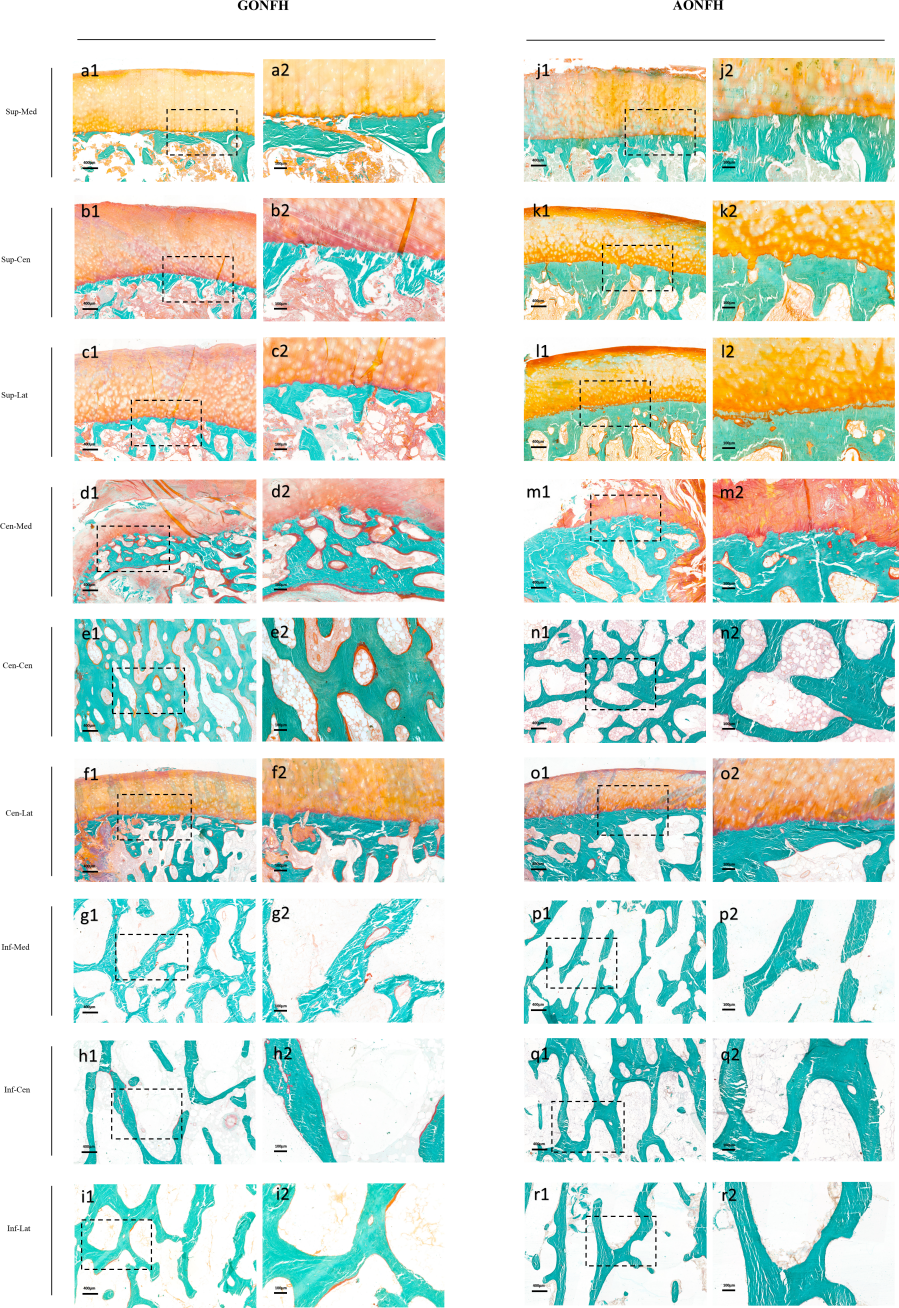
Photomicrographs of undecalcified sections of different ROIs in the femoral head from patients with GONFH **(A-I)** and patients with AONFH **(J-R)**. Cracks in bone matrix are artifacts which are produced during sectioning of the undecalcified bone specimen. Stain: Goldner's Trichrome; (a1-r1) magnification: ×40; (a2-r2) magnification: ×100.

In the necrotic-normal junction region, including Cen-Med, Cen-Cen and Cen-Lat regions, all the bone remodeling parameters were significantly higher in GONFH, compared with AONFH (**Figure 6**; **Table 3**). There were higher values of O.Th, OV/BV, OS/BS and OS/BV, indicating a more active bone formation status. Bone resorption activity was also higher, as suggested by higher values of erosion parameters including ES/BS, ES/BV and ES/TV.

**Table 3:** Comparison of bone remodeling parameters in the reactive interface (Cen-Med region, Cen-Cen region, Cen-Lat region) and normal region (Inf-Med region, Inf-Cen region, Inf-Lat region) between GONFH and AONFH.

Region	Variables	GONFH	AONFH	P	Region	Variables	GONFH	AONFH	P
Cen-Med	O.Th (μm)	16.730±10.190	12.771±6.945	**0.0354**	Inf-Med	O.Th (μm)	7.359±6.433	4.049±1.249	**0.0003**
	OV/BV (%)	4.815±1.935	2.847±2.412	**<0.0001**		OV/BV (%)	1.310±0.440	0.896±0.173	**<0.0001**
	OS/BS (%)	39.701±19.980	27.140±23.730	**0.0002**		OS/BS (%)	10.301±4.318	6.679±2.650	**<0.0001**
	OS/BV (mm^2^/m^3^)	3.737±1.521	2.309±1.829	**<0.0001**		OS/BV (mm^2^/mm^3^)	1.844±0.733	1.495±0.364	**0.0012**
	ES/BS (%)	14.810±2.272	9.278±1.832	**<0.0001**		ES/BS (%)	6.152±0.821	4.562±0.570	**<0.0001**
	ES/BV (mm^2^/mm^3^)	1.539±0.558	0.876±0.284	**<0.0001**		ES/BV (mm^2^/mm^3^)	1.242±0.675	1.125±0.379	0.2913
	ES/TV (mm^2^/mm^3^)	0.648±0.369	0.342±0.188	**<0.0001**		ES/TV (mm^2^/mm^3^)	0.392±0.208	0.311±0.090	**0.0138**
Cen-Cen	O.Th (μm)	19.770±12.690	11.601±6.160	**<0.0001**	Inf-Cen	O.Th (μm)	7.444±4.564	4.750±2.013	**<0.0001**
	OV/BV (%)	4.436±2.104	1.706±1.112	**<0.0001**		OV/BV (%)	1.215±0.168	0.909±0.117	**<0.0001**
	OS/BS (%)	42.170±31.940	19.990±15.141	**<0.0001**		OS/BS (%)	11.640±4.671	7.528±3.327	**<0.0001**
	OS/BV (mm^2^/m^3^)	4.178±5.389	1.596±1.082	**<0.0001**		OS/BV (mm^2^/mm^3^)	1.787±0.751	1.502±0.341	**0.0188**
	ES/BS (%)	14.860±3.297	9.524±1.650	**<0.0001**		ES/BS (%)	6.069±0.452	4.618±0.875	**<0.0001**
	ES/BV (mm^2^/mm^3^)	1.481±0.618	0.855±0.320	**<0.0001**		ES/BV (mm^2^/mm^3^)	1.026±0.474	1.012±0.358	0.8881
	ES/TV (mm^2^/mm^3^)	0.572±0.292	0.326±0.110	**<0.0001**		ES/TV (mm^2^/mm^3^)	0.399±0.213	0.343±0.108	0.3274
Cen-Lat	O.Th (μm)	17.550±10.691	12.140±4.949	**0.0184**	Inf-Lat	O.Th (μm)	6.868±4.469	3.841±1.366	**<0.0001**
	OV/BV (%)	5.288±3.022	2.028±1.297	**<0.0001**		OV/BV (%)	1.308±0.202	0.934±0.128	**<0.0001**
	OS/BS (%)	42.910±21.341	20.371±14.410	**<0.0001**		OS/BS (%)	9.772±3.753	6.810±2.567	**<0.0001**
	OS/BV (mm^2^/m^3^)	3.941±1.428	1.757±1.115	**<0.0001**		OS/BV (mm^2^/mm^3^)	1.813±0.861	1.586±0.379	0.1289
	ES/BS (%)	15.410±3.001	9.692±1.855	**<0.0001**		ES/BS (%)	6.105±0.443	4.784±0.858	**<0.0001**
	ES/BV (mm^2^/mm^3^)	1.586±0.643	0.935±0.364	**<0.0001**		ES/BV (mm^2^/mm^3^)	1.208±0.550	1.216±0.386	0.9348
	ES/TV (mm^2^/mm^3^)	0.635±0.202	0.362±0.145	**<0.0001**		ES/TV (mm^2^/mm^3^)	0.366±0.223	0.307±0.085	0.3173

Results are expressed as mean ± SD. Bold indicates statistically significant difference.

In Inf-Med region, most bone remodeling parameters were significantly higher in GONFH than AONFH, with the exception of ES/BV (**Figure 6**; **Table 3**). In Inf-Cen region, the bone formation parameters O.Th, OV/BV, OS/BS and OS/BV were significantly elevated in GONFH samples than those in AONFH (**Figure 6**; **Table 3**). The bone resorption parameter ES/BS was significantly elevated in GONFH. No significant differences were observed in ES/BV and ES/TV. In Inf-Lat region, there were higher values of O.Th, OV/BV, OS/BS in GONFH, indicating a more active bone formation status (**Figure 6**; **Table 3**). OS/BV were also higher in GONFH, although no significant difference was observed. The bone resorption parameter ES/BS was significantly elevated in GONFH. No significant differences were observed in ES/BV and ES/TV.

In order to assess how the gender difference of the subjects influence the results, we compared the differences of microstructural and bone remodeling parameters in the male patients between the two groups separately. The corresponding results were shown in the **Supplementary Tables 2**, **3**, showing similar trend in the male patients: GONFH exhibited a less sclerotic microarchitecture and a more active bone metabolic status in both the reactive interface and normal region. Accordingly, the gender difference of the subjects did not influence the final conclusion in the current study.

## Discussion

In this study, we analyzed simultaneously bone microarchitecture, histopathologic alterations and bone remodeling levels in different regions of the involved femoral head between GONFH and AONFH. The two groups shared similar microarchitectural characteristics in the necrotic region, whereas both the reactive interface and normal regions illustrated significant differences in the microstructure and histomorphometry. The reactive interface and normal regions revealed a less sclerotic microarchitecture, but a higher bone remodeling level in GONFH as compared with AONFH. Despite similar necrotic pathological manifestations, subchondral trabecular microfracture and debris was more severe in the necrotic region, and the reactive interface was more vascular in GONFH.

Microstructure is an important component of bone quality, and its integrity contributes to the maintenance of bone strength (12, 41). The microarchitectural patterns in different regions of the joint may differ (42), reflecting different types and levels of biomechanical loads (43, 44). The primary compressive trabeculae, which have a compact microarchitecture and great biomechanical strength, bear the majority of stress on the femoral head during daily activities (45). The microarchitectural analysis based on the three-pillar structure will deepen our understanding of the detailed overall morphological alterations of the entire femoral head in the development of ONFH and aid clinicians in reevaluating the disease’s severity, which may alter the indication for joint-preservation surgery for ONFH. Our results revealed that the reactive interface and normal region displayed a less sclerotic microarchitecture in GONFH as compared with AONFH, exhibiting a lower bone volume fraction, less trabecular number, and wider trabecular space, especially in the Cen-Cen region. The deteriorated microarchitecture has been reported to play a pivotal role in the failure of mechanical stabilization in the femoral head, and eventually leads to the progression of collapse (46). The alterations of microstructure may be due to the distinct bone remodeling status (47), which could be influenced by different reagents that affect bone metabolism such as glucocorticoid and alcohol.

Despite the observation of a reduced sclerotic microarchitecture, our investigation revealed that the reactive interface and normal region in GONFH exhibited higher bone remodeling levels. This result may be attributed to the potential that the activity of osteoclast-mediated bone resorption in GONFH was significantly higher, hence outweighing the effect of the increased bone production activity. This difference could be related to the varied biological reactions of relevant bone cells attempting to heal necrotic tissue (48), reflecting the unique consequences of these two causes. It has been postulated that glucocorticoid and alcohol impair femoral artery perfusion by mechanisms including vascular endothelial damage and microvascular thrombosis (8, 49). They can also induce the formation of intramedullary fat, which increases the pressure in the bones, leading to venous congestion and blocked arteries (10, 50, 51). Glucocorticoid has been reported to reduce the production of osteoblasts (52), increase the apoptosis of osteoblasts and prolong the life of osteoclasts (13, 53). Accumulating evidence has also shown that heavy alcohol consumption impairs bone homoeostasis, which disturbs the osteoblast formation and proliferation, and promotes osteoclastogenesis (54, 55). Despite the adverse effect on bone metabolism by both glucocorticoid and alcohol, glucocorticoid might illustrate a more negative interference on osteogenesis and reparative process in the condition of ONFH.

Both GONFH and AONFH showed a large continuous area of bone marrow necrosis, osteocytic death and disordered subchondral trabecular structure in the necrotic region, with a fibrous reactive interface at the boundary of the sequestrum. Chernetsky et al. found that histologic alterations of necrosis and repair evolved in a given sequence after bone death, the rate of which was faster in the GONFH (16). The similar histologic characteristics of necrosis between these two subgroups of ONFH were consistent with Chernetsky et al.’s earlier investigation (16). In the necrotic region of GONFH, the microstructure and organization of subchondral trabeculae were more severely compromised, and the vascularized fibrous interface was more prominent. The highly degraded and disordered subchondral microarchitecture could be ascribed to the biomechanically poor strength of trabeculae and increased osteoclast activity (56), both of which are associated with chronic overdose use of glucocorticoid under weight-bearing conditions. Angiogenesis is a crucial component of the reparative response after necrosis. In spite of glucocorticoid’s deleterious effects on bone angiogenesis in the pathogenesis of osteonecrosis, introduction of glucocorticoid could stimulate angiogenesis under certain conditions (57), which may explain the highly vascularized fibrous interface in GONFH. More research is necessary to understand the underlying mechanism. Alcohol in high concentrations has been shown to have a negative impact on neovascularization (58, 59), which explains why there is less vascularization visible in the reactive interface in AONFH.

The progression of collapse is influenced by the metabolic activities in the reaction interface, especially bone resorption of necrotic region (60). The activity of osteoclasts in the reactive interface exceeds that of osteoblasts, which may result in the more deteriorated microarchitecture, weakened mechanical strength and eventual collapse of the femoral head (61). For patients with GONFH, specific medical treatment at the early stage of the disease may help delay the progression of collapse by reducing the activity of osteoclasts at the reactive interface, especially for those patients with osteoporosis. Collectively, personalized treatment may bring greater benefits to ONFH patients with different etiologies.

There are limitations in this study. The lack of healthy individuals was one of the study’s limitations. Without healthy controls, all of these bone samples were taken from ONFH patients. The relatively young age of these patients undergoing THA makes the acquisition and age matching of control samples difficult. But for all that, we cannot reveal whether there was a specific degree of heterogeneity in the microstructure of the femoral head in ONFH patients, even with the same trend in healthy controls. Second, this was a cross-sectional study with femoral head specimens that lacked parameters of dynamic bone remodeling. *In vivo* micro-CT scanning of the hip joint is not possible.

Future developments in CT and MRI with enhanced spatial resolution may aid in the resolution of this issue (62–64).

## Conclusion

In summary, GONFH and AONFH showed similar microarchitecture characteristics in the necrotic region, while the reactive interface and normal region exhibited a less sclerotic microarchitecture and a more active remodeling status in GONFH than that in AONFH. In the aspect of histopathology, microfracture of subchondral trabeculae in the necrotic region was more severe and vasculature of the reactive interface was more abundant in GONFH. These phenomena might be due to the distinct biological effects of glucocorticoid and alcohol on bone metabolism and angiogenesis. This study not only provides valuable data from the microstructure and histomorphology aspects to guide the medication of patients with GONFH, but also helps to modify the surgical techniques in patients receiving joint-preserving therapy, especially concerning the debridement area related to the necrotic region and reactive interface.

## Data availability statement

The original contributions presented in the study are included in the article/**Supplementary Material**. Further inquiries can be directed to the corresponding author.

## Ethics statement

The studies involving human participants were reviewed and approved by Human Research Ethics committee of Shanghai Jiao Tong University Affiliated Sixth People’s Hospital. The patients/participants provided their written informed consent to participate in this study. Written informed consent was obtained from the individual(s) for the publication of any potentially identifiable images or data included in this article.

## Author contributions

All authors listed have read and approved all versions of the manuscript. YC had full access to all data in the study and took responsibility for the integrity of the data and the accuracy. Critical revision of the article for important intellectual content, YC, GL, KL, YM, BZ, FX, JY, JZ, CZ, and YF. All authors contributed to the article and approved the submitted version.
