# Supplementary material for: Less sclerotic microarchitecture pattern with increased bone resorption in glucocorticoid-associated osteonecrosis of femoral head as compared to alcohol-associated osteonecrosis of femoral head
Source: Front Endocrinol (Lausanne). 2023 Mar 8;14:1133674. doi: 10.3389/fendo.2023.1133674 (PMC10031038; doi:10.3389/fendo.2023.1133674)
Supplement: Supplementary file 1 [file Table_1.docx]

RMSE of different ROIs in glucocorticoid-associated ONFH

|  | Sup-Med | Sup-Cen | Sup-Lat | Cen-Med | Cen-Cen | Cen-Lat | Inf-Med | Inf-Cen | Inf-  Lat |
| --- | --- | --- | --- | --- | --- | --- | --- | --- | --- |
| BV/TV (%) | 3.954 | 4.706 | 4.457 | 4.878 | 3.235 | 4.313 | 3.391 | 4.713 | 4.161 |
| BS/BV(1/mm) | 1.095 | 0.856 | 0.920 | 0.781 | 0.332 | 1.022 | 1.330 | 1.049 | 1.162 |
| Tb.Th (μm) | 0.025 | 0.027 | 0.030 | 0.034 | 0.067 | 0.016 | 0.017 | 0.025 | 0.029 |
| Tb.N(1/mm) | 0.065 | 0.033 | 0.042 | 0.061 | 0.067 | 0.077 | 0.078 | 0.074 | 0.063 |
| Tb.Sp (μm) | 0.024 | 0.006 | 0.018 | 0.020 | 0.020 | 0.023 | 0.027 | 0.034 | 0.024 |
| Conn.D (1/mm^3^) | 0.299 | 0.966 | 0.358 | 0.380 | 0.179 | 0.673 | 0.235 | 0.269 | 0.260 |
| SMI | 0.259 | 0.615 | 0.311 | 0.348 | 0.256 | 0.357 | 0.176 | 0.317 | 0.256 |
| DA | 0.017 | 0.032 | 0.039 | 0.066 | 0.025 | 0.027 | 0.061 | 0.021 | 0.078 |

RMSE of different regions in alcohol-associated ONFH

|  | Sup-Med | Sup-Cen | Sup-Lat | Cen-Med | Cen-Cen | Cen-Lat | Inf-Med | Inf-Cen | Inf-  Lat |
| --- | --- | --- | --- | --- | --- | --- | --- | --- | --- |
| BV/TV (%) | 3.115 | 4.962 | 4.982 | 3.668 | 4.241 | 3.946 | 1.210 | 1.943 | 1.022 |
| BS/BV(1/mm) | 0.647 | 0.986 | 1.360 | 0.814 | 0.869 | 1.036 | 0.522 | 0.313 | 0.316 |
| Tb.Th (μm) | 0.010 | 0.029 | 0.026 | 0.019 | 0.019 | 0.027 | 0.013 | 0.031 | 0.018 |
| Tb.N(1/mm) | 0.071 | 0.058 | 0.107 | 0.159 | 0.121 | 0.120 | 0.111 | 0.296 | 0.266 |
| Tb.Sp (μm) | 0.072 | 0.021 | 0.044 | 0.013 | 0.070 | 0.069 | 0.037 | 0.045 | 0.046 |
| Conn.D (1/mm^3^) | 0.182 | 0.241 | 0.126 | 0.241 | 0.131 | 0.466 | 0.041 | 0.137 | 0.507 |
| SMI | 0.119 | 0.315 | 0.181 | 0.205 | 0.205 | 0.194 | 0.053 | 0.030 | 0.147 |
| DA | 0.064 | 0.034 | 0.077 | 0.175 | 0.129 | 0.025 | 0.087 | 0.130 | 0.250 |
