# Supplementary material for: Less sclerotic microarchitecture pattern with increased bone resorption in glucocorticoid-associated osteonecrosis of femoral head as compared to alcohol-associated osteonecrosis of femoral head
Source: Front Endocrinol (Lausanne). 2023 Mar 8;14:1133674. doi: 10.3389/fendo.2023.1133674 (PMC10031038; doi:10.3389/fendo.2023.1133674)
Supplement: Supplementary file 2 [file Table_2.docx]

**Supplementary Table 2** Comparison of microarchitecture parameters in male patients between GONFH and AONFH

| **Region** | **Variables** | **GONFH** | **AONFH** | **P** |  | **Region** | **Variables** | **GONFH** | **AONFH** | **P** |  | **Region** | **Variables** | **GONFH** | **AONFH** | **P** |
| --- | --- | --- | --- | --- | --- | --- | --- | --- | --- | --- | --- | --- | --- | --- | --- | --- |
| Sup-Med | BV/TV (%) | 22.95±8.68 | 27.81±13.35 | 0.176 |  | Cen-Med | BV/TV (%) | 38.45±17.54 | 49.48±15.76 | **0.007** |  | Inf-Med | BV/TV (%) | 16.01±8.16 | 19.55±9.99 | 0.102 |
|  | BS/BV(1/mm) | 13.68±3.58 | 12.96±3.57 | 0.400 |  |  | BS/BV(1/mm) | 10.17±3.80 | 8.29±2.91 | **0.018** |  |  | BS/BV(1/mm) | 16.14±4.17 | 14.45±3.89 | 0.082 |
|  | Tb.Th (μm) | 295.30±95.38 | 299.10±86.55 | 0.730 |  |  | Tb.Th (μm) | 402.90±161.60 | 453.60±173.70 | 0.176 |  |  | Tb.Th (μm) | 239.80±59.97 | 264.60±66.84 | 0.119 |
|  | Tb.N(1/mm) | 0.80±0.28 | 0.91±0.28 | 0.122 |  |  | Tb.N(1/mm) | 0.94±0.24 | 1.12±0.23 | **0.002** |  |  | Tb.N(1/mm) | 0.64±0.22 | 0.71±0.23 | 0.225 |
|  | Tb.Sp (μm) | 793.90±273.00 | 778.60±287.40 | 0.388 |  |  | Tb.Sp (μm) | 729.50±379.30 | 563.90±115.00 | **0.002** |  |  | Tb.Sp (μm) | 940.00±170.00 | 908.2±195.50 | 0.310 |
|  | Conn.D (1/mm^3^) | 3.36±1.55 | 3.50±1.38 | 0.691 |  |  | Conn.D (1/mm^3^) | 3.00±1.44 | 2.98±1.27 | 0.925 |  |  | Conn.D (1/mm^3^) | 2.22±0.72 | 2.31±0.95 | 0.959 |
|  | SMI | 1.65±0.59 | 1.34±0.76 | 0.097 |  |  | SMI | 0.79±1.08 | 0.16±1.00 | **0.003** |  |  | SMI | 1.71±0.39 | 1.61±0.46 | 0.322 |
|  | DA | 1.87±0.32 | 1.93±0.33 | 0.315 |  |  | DA | 1.71±0.36 | 1.78±0.36 | 0.431 |  |  | DA | 2.39±0.55 | 2.40±0.60 | 0.908 |
| Sup-Cen | BV/TV (%) | 30.20±13.14 | 33.94±12.33 | 0.221 |  | Cen-Cen | BV/TV (%) | 43.68±13.60 | 62.25±13.31 | **<0.0001** |  | Inf-Cen | BV/TV (%) | 34.96±5.34 | 37.30±11.58 | 0.3249 |
|  | BS/BV(1/mm) | 12.26±3.37 | 11.22±2.78 | 0.152 |  |  | BS/BV(1/mm) | 9.98±3.16 | 6.72±2.04 | **<0.0001** |  |  | BS/BV(1/mm) | 11.25±2.39 | 10.88±3.11 | 0.5933 |
|  | Tb.Th (μm) | 319.00±119.20 | 330.90±86.43 | 0.198 |  |  | Tb.Th (μm) | 375.60±136.20 | 521.80±184.80 | **<0.0001** |  |  | Tb.Th (μm) | 294.30±54.19 | 327.70±95.86 | 0.1377 |
|  | Tb.N(1/mm) | 0.95±0.26 | 1.03±0.27 | 0.186 |  |  | Tb.N(1/mm) | 1.18±0.15 | 1.25±0.20 | 0.151 |  |  | Tb.N(1/mm) | 1.07±0.12 | 1.14±0.19 | 0.0971 |
|  | Tb.Sp (μm) | 736.10±336.10 | 691.50±234.10 | 0.690 |  |  | Tb.Sp (μm) | 560.10±101.60 | 455.20±93.47 | **<0.0001** |  |  | Tb.Sp (μm) | 652.50±67.93 | 614.50±98.90 | 0.0522 |
|  | Conn.D (1/mm^3^) | 3.56±1.64 | 3.51±1.40 | 0.906 |  |  | Conn.D (1/mm^3^) | 4.00±1.95 | 2.65±1.37 | **0.001** |  |  | Conn.D (1/mm^3^) | 3.11±0.92 | 3.35±1.54 | 0.720 |
|  | SMI | 1.20±0.70 | 1.03±0.90 | 0.141 |  |  | SMI | 0.45±0.82 | -0.71±1.36 | **<0.0001** |  |  | SMI | 1.10±0.50 | 0.71±0.59 | **0.005** |
|  | DA | 1.94±0.43 | 1.79±0.33 | 0.261 |  |  | DA | 1.81±0.32 | 1.77±0.38 | 0.376 |  |  | DA | 2.55±0.41 | 2.52±0.45 | 0.796 |
| Sup-Lat | BV/TV (%) | 29.25±9.74 | 33.98±9.79 | 0.064 |  | Cen-Lat | BV/TV (%) | 45.14±16.48 | 54.49±14.09 | **0.011** |  | Inf-Lat | BV/TV (%) | 17.76±5.03 | 20.36±8.57 | 0.0998 |
|  | BS/BV(1/mm) | 12.92±3.74 | 11.88±3.79 | 0.191 |  |  | BS/BV(1/mm) | 9.09±3.46 | 7.39±2.36 | **0.013** |  |  | BS/BV(1/mm) | 16.61±4.23 | 14.94±3.66 | 0.077 |
|  | Tb.Th (μm) | 319.30±100.50 | 345.40±70.99 | 0.051 |  |  | Tb.Th (μm) | 425.30±158.80 | 494.20±174.00 | 0.077 |  |  | Tb.Th (μm) | 230.10±56.28 | 249.80±54.81 | 0.089 |
|  | Tb.N(1/mm) | 0.85±0.31 | 0.99±0.23 | **0.042** |  |  | Tb.N(1/mm) | 1.07±0.16 | 1.14±0.18 | 0.101 |  |  | Tb.N(1/mm) | 0.68±0.21 | 0.79±0.23 | **0.036** |
|  | Tb.Sp (μm) | 755.90±207.00 | 718.50±201.20 | 0.477 |  |  | Tb.Sp (μm) | 604.20±140.60 | 544.30±111.00 | **0.043** |  |  | Tb.Sp (μm) | 912.70±167.60 | 830.40±156.60 | 0.099 |
|  | Conn.D (1/mm^3^) | 3.43±1.66 | 3.08±0.96 | 0.548 |  |  | Conn.D (1/mm^3^) | 3.07±1.41 | 2.62±1.19 | 0.149 |  |  | Conn.D (1/mm^3^) | 2.53±0.77 | 2.89±1.28 | 0.503 |
|  | SMI | 1.58±0.52 | 1.05±0.67 | **0.003** |  |  | SMI | 0.33±1.05 | -0.14±1.20 | 0.077 |  |  | SMI | 1.65±0.40 | 1.55±0.41 | 0.279 |
|  | DA | 1.89±0.48 | 1.70±0.26 | **0.040** |  |  | DA | 1.76±0.31 | 1.77±0.34 | 0.983 |  |  | DA | 2.45±0.62 | 2.16±0.61 | **0.041** |

Results are expressed as mean ± SD. Bold indicates statistically significant difference.
