# Supplementary material for: Less sclerotic microarchitecture pattern with increased bone resorption in glucocorticoid-associated osteonecrosis of femoral head as compared to alcohol-associated osteonecrosis of femoral head
Source: Front Endocrinol (Lausanne). 2023 Mar 8;14:1133674. doi: 10.3389/fendo.2023.1133674 (PMC10031038; doi:10.3389/fendo.2023.1133674)
Supplement: Supplementary file 3 [file Table_3.docx]

**Supplementary Table 3**

Comparison of bone remodeling parameters in male patients between GONFH and AONFH

| **Region** | **Variables** | **GONFH** | **AONFH** | **P** |  | **Region** | **Variables** | **GONFH** | **AONFH** | **P** |
| --- | --- | --- | --- | --- | --- | --- | --- | --- | --- | --- |
| Cen-Med | O.Th (μm) | 15.71±10.13 | 12.79±7.02 | 0.1810 |  | Inf-Med | O.Th (μm) | 8.62±8.02 | 4.07±1.26 | **0.0001** |
|  | OV/BV (%) | 4.34±1.51 | 2.89±2.42 | **<0.0001** |  |  | OV/BV (%) | 1.36±0.58 | 0.90±0.18 | **<0.0001** |
|  | OS/BS (%) | 40.95±19.95 | 27.62±23.74 | **0.0015** |  |  | OS/BS (%) | 9.91±3.79 | 6.70±2.67 | **0.0004** |
|  | OS/BV (mm2/m3) | 3.64±1.17 | 2.35±1.83 | **0.0002** |  |  | OS/BV (mm2/mm3) | 1.69±0.62 | 1.50±0.37 | 0.0530 |
|  | ES/BS (%) | 14.45±2.20 | 9.28±1.85 | **<0.0001** |  |  | ES/BS (%) | 6.14±1.06 | 4.55±0.56 | **<0.0001** |
|  | ES/BV (mm2/mm3) | 1.43±0.50 | 0.87±0.28 | **<0.0001** |  |  | ES/BV (mm2/mm3) | 1.15±0.57 | 1.12±0.38 | 0.8050 |
|  | ES/TV (mm2/mm3) | 0.61±0.23 | 0.34±0.19 | **<0.0001** |  |  | ES/TV (mm2/mm3) | 0.38±0.21 | 0.31±0.09 | 0.1269 |
| Cen-Cen | O.Th (μm) | 19.16±12.42 | 11.61±6.22 | **0.0038** |  | Inf-Cen | O.Th (μm) | 7.96±5.48 | 4.76±2.03 | **0.0004** |
|  | OV/BV (%) | 4.32±1.93 | 1.73±1.11 | **<0.0001** |  |  | OV/BV (%) | 1.17±0.16 | 0.91±0.12 | **<0.0001** |
|  | OS/BS (%) | 45.74±38.83 | 20.34±15.10 | **0.0002** |  |  | OS/BS (%) | 12.08±5.11 | 7.57±3.35 | **<0.0001** |
|  | OS/BV (mm2/m3) | 5.21±4.17 | 1.62±1.08 | **<0.0001** |  |  | OS/BV (mm2/mm3) | 1.75±0.77 | 1.50±0.34 | 0.1080 |
|  | ES/BS (%) | 14.01±3.60 | 9.53±1.67 | **<0.0001** |  |  | ES/BS (%) | 6.06±0.52 | 4.63±0.88 | **<0.0001** |
|  | ES/BV (mm2/mm3) | 1.59±0.74 | 0.85±0.32 | **<0.0001** |  |  | ES/BV (mm2/mm3) | 0.96±0.43 | 1.01±0.36 | 0.6622 |
|  | ES/TV (mm2/mm3) | 0.59±0.33 | 0.33±0.11 | **<0.0001** |  |  | ES/TV (mm2/mm3) | 0.42±0.26 | 0.34±0.11 | 0.5839 |
| Cen-Lat | O.Th (μm) | 16.92±11.67 | 11.80±4.94 | 0.1922 |  | Inf-Lat | O.Th (μm) | 7.31±5.50 | 3.84±1.38 | **0.0001** |
|  | OV/BV (%) | 4.94±3.18 | 2.05±1.31 | **<0.0001** |  |  | OV/BV (%) | 1.32±0.22 | 0.93±0.13 | **<0.0001** |
|  | OS/BS (%) | 40.23±22.03 | 20.71±14.36± | **<0.0001** |  |  | OS/BS (%) | 9.40±4.04 | 6.87±2.56 | **0.0057** |
|  | OS/BV (mm2/m3) | 3.87±1.45 | 1.78±1.11 | **<0.0001** |  |  | OS/BV (mm2/mm3) | 1.78±0.92 | 1.59±0.38 | 0.5031 |
|  | ES/BS (%) | 14.55±2.58 | 9.69±1.87 | **<0.0001** |  |  | ES/BS (%) | 6.02±0.43 | 4.79±0.87 | **<0.0001** |
|  | ES/BV (mm2/mm3) | 1.65±0.80 | 0.92±0.36 | **<0.0001** |  |  | ES/BV (mm2/mm3) | 1.24±0.61 | 1.21±0.38 | 0.8212 |
|  | ES/TV (mm2/mm3) | 0.64±0.22 | 0.36±0.15 | **<0.0001** |  |  | ES/TV (mm2/mm3) | 0.37±0.27 | 0.31±0.09 | 0.7110 |

Results are expressed as mean ± SD. Bold indicates statistically significant difference.
