# Supplementary figures and images for: Less sclerotic microarchitecture pattern with increased bone resorption in glucocorticoid-associated osteonecrosis of femoral head as compared to alcohol-associated osteonecrosis of femoral head
Source: Front Endocrinol (Lausanne). 2023 Mar 8;14:1133674. doi: 10.3389/fendo.2023.1133674 (PMC10031038; doi:10.3389/fendo.2023.1133674)

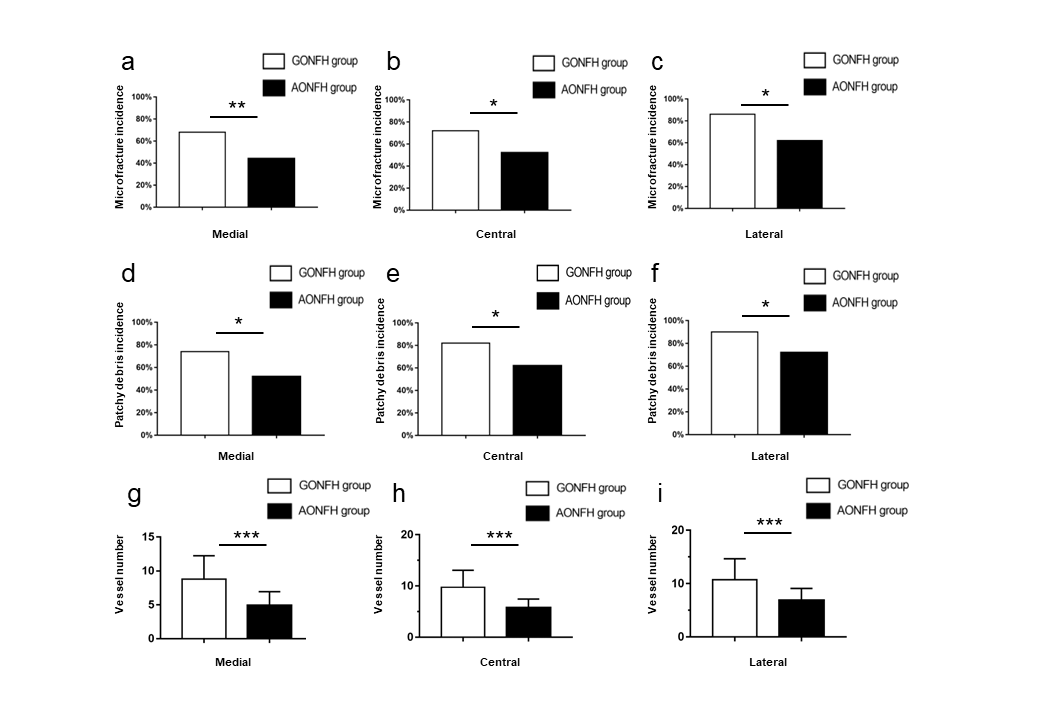

Supplement: Supplementary file 4 [file Image_1.tif]
